# Contribution of the Rise in Cohabiting Parenthood to Family Instability: Cohort Change in Italy, Great Britain, and Scandinavia

**DOI:** 10.1007/s13524-019-00823-0

**Published:** 2019-11-11

**Authors:** Elizabeth Thomson, Maria Winkler-Dworak, Éva Beaujouan

**Affiliations:** 1 Demography Unit, Department of Sociology, Stockholm University, 106 81 Stockholm, Sweden; 2 Center for Demography and Ecology, University of Wisconsin–Madison, 4412 Sewell Social Sciences, 1180 Observatory Drive, Madison, WI 53706 USA; 3 Vienna Institute of Demography/Austrian Academy of Sciences/Wittgenstein Centre for Demography and Global Human Capital, Campus/D5, Welthandelsplatz 2/Ebene 2, 1020 Vienna, WU Austria; 4 Department of Socioeconomics, Vienna University of Economics and Business/Wittgenstein Centre for Demography and Global Human Capital, Building D4, 3rd Floor, Welthandelsplatz 1, 1020 Vienna, Austria

**Keywords:** Cohabitation, Marriage, Separation, Divorce, Microsimulation

## Abstract

In this study, we investigate through microsimulation the link between cohabiting parenthood and family instability. We identify mechanisms through which increases in cohabiting parenthood may contribute to overall increases in separation among parents, linking micro-level processes to macro-level outcomes. Analyses are based on representative surveys in Italy, Great Britain, and Scandinavia (represented by Norway and Sweden), with full histories of women’s unions and births. We first generate parameters for the risk of first and higher-order birth and union events by woman’s birth cohort and country. The estimated parameters are used to generate country- and cohort-specific populations of women with stochastically predicted family life courses. We use the hypothetical populations to decompose changes in the percentage of mothers who separate/divorce across maternal birth cohorts (1940s to 1950s, 1950s to 1960s, 1960s to 1970s), identifying how much of the change can be attributed to shifts in union status at first birth and how much is due to change in separation rates for each union type. We find that when cohabiting births were uncommon, increases in parents’ separation were driven primarily by increases in divorce among married parents. When cohabiting parenthood became more visible, it also became a larger component, but continued increases in parents’ divorce also contributed to increasing parental separation. When cohabiting births became quite common, the higher separation rates of cohabiting parents began to play a greater role than married parents’ divorce. When most couples had their first birth in cohabitation, those having children in marriage were increasingly selected from the most stable relationships, and their decreasing divorce rates offset the fact that increasing proportions of children were born in somewhat less stable cohabiting unions.

## Introduction

Intimate partnerships in affluent societies have undergone dramatic changes since the mid-twentieth century. Couples have increasingly started living together without the legal commitment of marriage and have become increasingly likely to separate if they marry (Bumpass et al. 1991; Härkönen and Dronkers 2006; Kennedy and Ruggles 2013, 2014; Manning et al. 2014; Raley and Bumpass 2003; Sobotka and Toulemon 2008). These trends are also found among parents; increasing proportions of adults have their first child in cohabitation, and separation or divorce is increasingly likely to follow (Beaujouan 2016; Bernardi and Martinez-Pastor 2011; Musick and Michelmore 2015). Parental separation at the least creates temporary dislocations in family life and relationships, and at worst produces more deleterious outcomes for children (Thomson and McLanahan 2012). Increases in the proportion of parents and children experiencing separation are therefore of societal concern.

Because nonmarital cohabitation signals a weaker commitment to the relationship and to a particular partner than does marriage (Perelli-Harris and Bernardi 2015; Perelli-Harris et al. 2014), it can be viewed as a precursor at the individual level of the likelihood of separation. Thus, having a child in cohabitation could be viewed as a pathway to parents’ eventual separation. Empirical evidence is clear that those who become parents in cohabitation are more likely to separate than those who married before having children (Andersson and Philipov 2002; Andersson et al. 2017; Goodman and Greaves 2010; Heuveline et al. 2003; Schnor 2014). The spread of cohabiting parenthood of the last decades may thus have produced overall increases in separation rates among parents.

Links between changes in individual-level behavior and changes at the population level are not, however, typically so straightforward given the complexity of individual-level interactions. Understanding links at the population level requires an examination of not only mechanisms through which individual processes (such as partnering and childbearing) are linked over the life course but also those through which the aggregation of individual-level behavior shapes macro-level change (Billari 2015).

In this study, we use microsimulation that enables aggregate inferences from micro behavioral changes while taking into account the heterogeneity among individuals and the complexity of individual processes (Billari 2006; Matysiak and Vignoli 2009). We develop arguments about the macro-level implications of increasing births in cohabitation for levels of separation after first birth, and then test their validity on simulated family life courses generated from nationally representative survey data from Italy, Great Britain, and Scandinavia (represented by Norway and Sweden). We decompose changes across cohorts in separation after first birth into changes associated with shifts in union status at birth and changes in separation rates^1^ among parents who were cohabiting versus married at first birth. To further investigate the implication of compositional shifts for aggregate levels of parental separation, we compare results for national contexts with quite different levels and shifts over time in cohabiting parenthood and parental separation.

## Micro- to Macro-Level Links Between Cohabitation and Parental Separation

At the micro level, cohabitation is by design a less stable form of intimate partnership than marriage. Most couples choose cohabitation precisely because it requires a lower level of commitment than marriage and offers the opportunity to test their relationship for the greater commitment of marriage (Perelli-Harris and Bernardi 2015; Perelli-Harris et al. 2014). Many relationships put to the test will fail, producing a higher rate of separation than for couples who go on to marry or marry without cohabiting.

It is not obvious, however, that parents who cohabit should be less committed to each other than parents who have married. Poortman and Mills (2012) argued that relationship commitment—whether through marriage or not—precedes partners’ willingness to undertake the structural and moral commitments of parenthood. Parenthood generates a shared commitment of economic, social, and emotional resources to a common child. Shared responsibilities to children also make the process of dissolution much more difficult and perhaps equal to the difficulties of divorce (Perelli-Harris and Sánchez Gassen 2012; Sánchez Gassen and Perelli-Harris 2015). Thus, parenthood may be considered by many as providing the same signal as marriage about commitment to the partner relationship (Perelli-Harris and Bernardi 2015).

Despite these considerations, the micro-level association between cohabiting parenthood and separation is positive across a broad range of societies (Andersson and Philipov 2002; Andersson et al. 2017; Heuveline et al. 2003; Kiernan 1999; Musick and Michelmore 2018; for country-specific studies, see the review in Schnor 2014). The association may be due to the still more fragile nature of cohabitation. Couples may “slide” into cohabitation without making a deliberate decision to spend their lives together (Manning and Smock 2005; Sassler 2004; Stanley et al. 2006). In the same way, cohabiting couples could slide into parenthood more often than married couples. In the United States, for example, cohabitors are more likely than married couples to have unplanned pregnancies and births (Hayford and Guzzo 2010; Lichter et al. 2016).

The naïve implication of this well-established micro-level relationship is that increases in cohabiting births should lead to increases in parental separation. Relationships observed at the individual level are not, however, always observed at the aggregate level. Changes in a population outcome (parental separation) may result from changes in the composition of the population (cohabiting and married parents), changes in the propensities across groups to experience the event (to separate), or some combination of the two.

To understand the compositional effect, assume that the growing group of people who have their children in cohabitation instead of marriage adopt systematically the behavior (separation rates) of the original smaller group having their first child in cohabitation. A growth in the share of cohabiting parents—who separate at a rate that is larger than that for married parents—would then induce a rise in the overall separation rate among parents. Of course, the assumption is extreme: although several theoretical arguments have been made for the experience of cohabitation as a cause of instability, the only natural experiment on “just in case” marriages found no such effect (Holland et al. 2017).

We thus expect in addition a rate effect. As the group of cohabiting parents grows, it becomes less different from the shrinking group of married parents. Cohabitation becomes a weaker signal of the underlying stability of the relationship, so separation rates of cohabiting parents become closer to those of married parents. Empirical evidence is quite strong for a narrowing gap in stability as cohabiting parenthood becomes more common (Andersson et al. 2017; Clarke and Jensen 2004; Pelletier 2016; Schnor 2014; but see Jensen and Clausen 2003). On the other hand, suppose there has been an upward shift in the threshold of relationship quality and commitment required for marriage but not for parenthood. In that case, among all parents, the gap between those who were married versus cohabiting at first birth could grow, and the increase in cohabiting births would generate higher rates of parental separation overall.

Our study therefore distinguishes composition and rate effects on the macro level as sources of change in overall parental separation risks. We further disaggregate the composition and rate effects by categories of union status (cohabitation vs. marriage) at birth. To date, no such study exists, and microsimulation is the perfect tool for such estimations.

## Using Microsimulation to Disentangle the Link Between Cohabiting Parenthood and Parental Separation at the Population Level

Evidence on the mechanisms that might link the rise in cohabiting parenthood to the rise in parental separation is limited. Musick and Michelmore (2015) used the 1995 and 2006–2010 U.S. National Surveys of Family Growth to estimate the risk of separation for couples having a first shared birth within 10 years of the interview. Between the surveys, the proportion of such births in cohabitation increased from 17 % to 35 %. Their analysis derived probabilities of separation within five years of birth from the estimated monthly hazard rates, incorporating indicators of cohabitations and marriages prior to first birth. With the characteristics of cohabiting and married parents in the earlier period held constant, the increase in cohabiting births contributed to an increase of only 1 percentage point (15 % to 16 %) in the likelihood of parents separating within five years. Changes in the relative risks of separation by union status predicted a *decrease* in overall probability of separation from 17 % to 14 %.

Rackin and Gibson-Davis (2018) applied Kitagawa’s (1955) decomposition method to quantify the contribution of cohabiting parenthood to the number of family transitions (including, but not limited to, separation) experienced by firstborn children up to age 5. The increasing prevalence of maternal cohabitation (composition) was more important than changing rates in contributing to increases in number of transitions across time. Although transitions included separation of the child’s parents, they also included entry into a cohabiting versus married stepfamily, thereby making it difficult to distinguish contributions to parental separation per se.

The setup of a decomposition using observed data encounters several difficulties. First, a decomposition requires large-scale data sources, which are rare. Whereas official statistics make available only total divorce rate and births out of marriage, population-based sample surveys are required to link birth and union histories (Andersson and Philipov 2002; Andersson et al. 2017; Bumpass and Lu 2000; Bumpass and Raley 1995; Heuveline et al. 2003; Kennedy and Bumpass 2008; Kiernan 1999; Perelli-Harris et al. 2010b). Limited sample sizes generate instability in estimates of births and separation. In addition, a critical issue in linking aggregate levels of cohabiting births to those of parental separation is that births as well as separation can occur over a period of years after union formation. For that reason, a cohort rather than period approach is favored, allowing women to form unions, have children, and/or separate at different ages and therefore in different periods.

Microsimulation provides a solution for most of these concerns and limitations. It not only deduces from the individual life-course transitions the associated aggregate outcomes but also allows for tracing out the evolution of family states over the life course (Aassve et al. 2006). It is particularly useful when sample sizes in observational studies are small and associations are masked or distorted by sharp fluctuations. A further benefit of microsimulation is that it provides estimates of complete family life courses for cohorts who are still of reproductive age at the time of the survey. Estimated hazard rates of single processes (birth and union transitions) by age from the observed sample can be used to simulate family life courses from ages 15 to 50 for all cohorts. One thereby obtains simulated family events at older ages for the younger cohorts not yet observed through the older ages. The hazard models can be viewed as an engine that links earlier life course events and statuses to later-life transitions and statuses. The simulated population is the distribution of specific life courses that is produced by that engine.

## Cohabiting Parenthood and Parental Separation in Italy, Great Britain, and Scandinavia

**Fig. 1:**
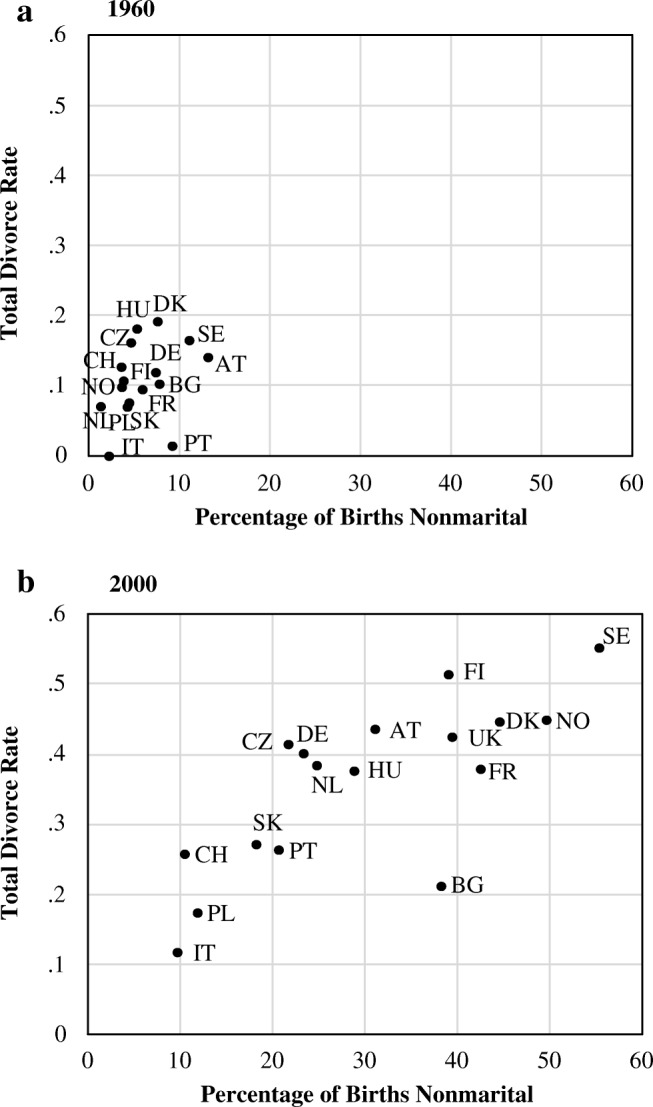
Total divorce rate and percentage of births out of marriage

The relationship between cohabiting parenthood and parental separation likely varies over time and place. Particularly, the country economic and cultural underpinnings differ, and separation and births in cohabitation emerged at very different times and at diverse rhythms. National statistics do not provide trend data on cohabiting parenthood and parents’ separation but rather only on related indicators: nonmarital births and divorce. Both have dramatically increased in most countries, beginning in the 1970s in northern and western Europe, a decade or so later in eastern and central Europe, and even later in southern Europe (Klüsener et al. 2013; Sobotka and Toulemon 2008). Figure 1 shows that in 1960, nonmarital births and divorce rates were low in most countries, less than 13 % of all births for the former and less than 20 % of marriages for the latter. By 2000, both rates had dramatically increased, and a clear positive association between them developed.

The increase in nonmarital births is a good proxy for increases in cohabiting parenthood; several studies have shown that most of the increase in nonmarital childbearing has arisen from increases in births to cohabiting parents rather than to mothers living alone (Kennedy and Bumpass 2008; Perelli-Harris et al. 2010b; Thomson and Eriksson 2013). The same is not true for divorce and parental separation. As increasing proportions of the population cohabit and separate without having ever been registered as a couple through marriage, divorce rates have become an increasingly poor indicator of union stability (Andersson and Philipov 2002; Andersson et al. 2017; Kennedy and Ruggles 2014; Raley and Bumpass 2003). Unfortunately, very little data exist on increases in parental separation and divorce.

We consider three societal contexts with different levels and histories of cohabiting parenthood and parental separation. Italy represents what is often termed the *southern European family pattern*: late home-leaving and family formation and very low rates of cohabitation and divorce. Nonmarital births increased from less than 5 % in 1960 to only 10 % by 2000 but have since more than doubled (Klüsener 2015). Almost all the increase was composed of births to cohabiting women; survey estimates show the percentage of births to cohabiting women increasing from 1 % in the 1970s to as much as 10 % in the early 2000s, still among the very lowest in Europe (Andersson and Philipov 2002; Andersson et al. 2017; Perelli-Harris et al. 2010b). The total divorce rate increased somewhat between 1970 and 1990 but also remained by far the lowest in Europe (Sobotka and Toulemon 2008). Estimates for parental separation are available only for the early 1990s and early 2000s, increasing between periods but remaining quite low in comparative perspective, at 13 % before a child’s 15th birthday (Andersson and Philipov 2002; Andersson et al. 2017).

In Great Britain, nonmarital childbearing began to increase around 1970 and then exploded to almost 50 % of births in the early 2000s (Klüsener 2015). Most but not all of the increase was due to cohabiting births—from 2 % in the 1970s to 31 % in the early 2000s (Perelli-Harris et al. 2010b). The crude divorce rate almost quadrupled between 1970 and 1985 and then leveled off at relatively high levels (Sobotka and Toulemon 2008). Trend data for separation or divorce among parents have not been generated for Great Britain.

Sweden and Norway, representing Scandinavia, have been forerunners in family change. In 1960, less than 10 % of births occurred out of marriage in both countries. Subsequent dramatic increases began in the 1970s, with Sweden leading Norway by about five years (Klüsener 2015). By the early 2000s, 55 % of births in both countries were nonmarital. Virtually all the increase was due to births in cohabitation (Andersson and Philipov 2002; Andersson et al. 2017; Perelli-Harris et al. 2010b; Thomson and Eriksson 2013) so that Sweden and Norway continue to lead in the percentage of births to cohabiting couples. The crude divorce rates in Sweden and Norway more than doubled in the mid-1970s, with a smaller increase during the 1990s, remaining among the highest in Europe (Sobotka and Toulemon 2008). Until the latest period, Swedish divorce rates were somewhat higher than those in Norway. In Sweden, about 15 % of children born to cohabiting or married parents experienced their parents’ separation by age 15, increasing to 35 % by the late 1990s (Thomson and Eriksson 2013). In both Sweden and Norway, the likelihood of parental separation remained stable at about the same level in the early 2000s, still among the highest in Europe (Andersson et al. 2017).

Taken together, these three contexts provide theoretically useful contrasts in family change. The forerunners were Sweden and Norway, starting from relatively low levels of cohabiting parenthood and parental separation in 1960 and experiencing dramatic upswings in the 1970s and 1980s. Most recently, both countries appear to be leaders in a leveling off at what may be maximum levels for Europe.^2^ By contrast, Italy provides the tail end of family change, having virtually no nonmarital births or divorce until the 1990s. By the early 2000s, cohabiting parenthood and parental separation had reached levels found in Scandinavia more than four decades earlier. Great Britain provides a middle case, also starting from a very traditional position in the 1960s, and experiencing the explosion in nonmarital births later than the Scandinavian countries. Because a significant part of this increase was births to mothers living alone, cohabiting parenthood remained well below the Scandinavian levels. Divorce increased over the same periods in Great Britain and Sweden but remained higher in Sweden (Sobotka and Toulemon 2008).

In terms of the decomposition discussed earlier, we expect that cohabiting parenthood played a limited role in separation of Italian first-time parents (no composition effect), with changes in divorce rates having made the greatest contribution, at least until the most recent cohorts. In Great Britain, changes in divorce rates are likely also to have been the primary early driver of increases in parental separation, whereas cohabiting parenthood is expected to have increased its contribution over time with no leveling off. In the Scandinavian context, we expect that the early increases in cohabiting parenthood played a compositional role in separations of childbearing unions, but that contributions were much reduced because cohabiting births were becoming as common as marital births.

## Data

We analyzed synthetic family life courses representative of each country. They were generated in microsimulations parameterized from Italian, British, and Scandinavian survey data with birth and union histories dated by year and month. For Italy, we used the multipurpose household surveys on Family and Social Subjects, carried out in 2003 and 2009. The 2003 survey constitutes the Italian GGS survey, so we used the version of the histories that had been harmonized with other GGS surveys by the Nonmarital Childbearing Network (Perelli-Harris et al. 2010a; www.nonmarital.org). We made small corrections on union order in an earlier version of the Harmonized Histories^3^ and also harmonized the 2009 data to correspond. We selected women born in Italy in 1940 or later, who had their first child or entered a partnership, if any, after age 15 (*N* = 30,255).

Estimations for Great Britain were based on 10 data sets (2000–2009) from the Centre for Population Change GHS database 1979–2009 (see Beaujouan et al. 2014 for details) and the 2009 wave of the Understanding Society Survey. The validity of partnership histories is good in the GHS series (Berrington et al. 2011), and checks in the Understanding Society surveys also correspond well with official statistics (Winkler-Dworak et al. 2019). The birth histories in the GHS had been adjusted and the series had been reweighted because children in the household were not always reported (Beaujouan et al. 2011; Ní Bhrolcháin et al. 2011). In our final sample, completed fertility levels matched closely the numbers from vital statistics (Winkler-Dworak et al. 2019). The final sample consisted of 61,718 women who were born in Great Britain in 1940 or later and had their first child or entered a partnership, if at all, after age 15, as for Italy.

We combined harmonized versions of the 2007/2008 Norwegian and 2012/2013 Swedish GGSs. The Norwegian histories had been validated for cohorts born since the mid-1940s (Vergauwen et al. 2015); we used Swedish administrative registers to validate a number of parameters in the simulated population (Winkler-Dworak et al. 2019). Both surveys were based on random samples taken from population registers and were carried out with a combination of computer-assisted telephone interviews and postal questionnaires. Each survey had a smaller sample than for Italy or Great Britain; by combining the samples, we were able to make distinctions in union and birth histories that would not have been possible with the separate samples. Differences between the two countries in birth and union behaviors were observed but were much closer than to the other countries (e.g., Andersson et al. 2017). We applied the same selection criteria as in Italy and Great Britain, producing an analytic sample of 6,589 Norwegian-born women and 4,446 Swedish-born women, for a total of 11,035 women.

## Methods

Our goal was to decompose changes in separation among parents on the macro level into its several components: (1) changes in the union status of first births and (2) changes in separation rates among parents with different union status at first birth. As noted earlier, the decompositions were performed on a population of simulated life courses rather than the life courses observed in the three data sets.

We estimated hazard models from the observed data for the first through fourth live birth and union events, separately for the first such event and any higher-order event: cohabitation or direct marriage (competing risk); separation or marriage (competing risk) from a cohabiting union; and divorce. Piecewise constant exponential models included age; birth cohort; age of youngest child; and detailed combinations of prior unions and births, including distinctions between births with previous or current partners. They also included several duration–cohort interactions with stepwise functions to represent linear splines. Model specifications, statistical methods, and parameter estimates are provided in Winkler-Dworak et al. (2019).

In the samples, each transition probability—conditional on earlier transitions—is of course estimated with error. The microsimulation takes into account the error component to generate the most likely distribution of life courses in a population that would reproduce the observed set of transition probabilities. Further details of the microsimulation can be found in Winkler-Dworak et al. (2019).

We used the mother’s birth cohort as the basis for defining change in cohabiting births and parental separation. We did not estimate change in parental separation from the perspective of children because the family life trajectories available in the survey are those of women. The union experiences prior to birth that underlie subsequent elements of shifts in family life trajectories across cohorts are not representative of the population of children. Furthermore, because of the very large delay in age at first birth across the maternal birth cohorts we observed, we specified cohort-specific duration dependence for first births in order to appropriately model the family life courses. Given large shifts in the timing of family events across cohorts, we would not necessarily be able to replicate observed change in parental separation by year of the child’s birth as we could do for maternal birth cohorts.

Younger cohorts in the surveys are not observed through the end of their childbearing years. Because of the proportional hazard assumption, we could estimate hazard rates for all ages and for all cohorts, conditioned on having the same partnership and childbearing history prior to the end of observation—that is, for events at ages observed only for older cohorts.^4^ Although we included all cohorts in the hazard regressions, we present simulated family life trajectories only for cohorts who could be observed until at least age 30—that is, born before 1980 in the original data sets.

For each country and birth cohort, 1 million synthetic family life courses (birth and union histories) were simulated. We simulated the family life trajectories for Sweden and Norway separately from models that distinguished the main country effect and for some events (depending on model fit), a country-cohort interaction. The two simulated populations were weighted in relation to the number of women observed in each sample and were then combined to form a single Scandinavian population.

We compared estimates of several demographic parameters from the simulated populations with the observed samples and with available administrative statistics from each country. Simulated indicators replicated very closely those observed in the survey samples and/or national statistics. (For further details on the validation of the microsimulation results, see Winkler-Dworak et al. 2019.)

From the simulated populations, we selected women who had a first birth in a cohabiting or marital union prior to age 40, and we estimated the risk of separation from the father of their first child, up to age 50 or the youngest child’s age 16, whichever comes first. We compared separation probabilities in the synthetic cohorts for the populations that were cohabiting versus married at the time of the first birth, by cohort and country. We applied standardization and decomposition methods (Kitagawa 1955) to derive standardized rates and standardized composition values for each cohort change (1940s to 1950s, 1950s to 1960s, and 1960s to 1970s), taking average cell composition and average cell rates, respectively, as weights. To simultaneously contrast multiple populations (birth cohorts), we combined standardization results from all pairwise combinations of cohorts to obtain composite standardized rates and composition values (cf. Das Gupta 1993:197, chapter 6). We also applied methods developed by Chevan and Sutherland (2009) to generate a secondary decomposition of the standardized rates and composition values into components by categories of union status at first birth.

## Results

The hazard estimates used to microsimulate family life trajectories (Winkler-Dworak et al. 2019) confirmed many well-known relationships among union formation, union dissolution, and fertility. Those most relevant for our research question are that births were more likely during marriage than during cohabitation and even more so than when not in a coresidential union. Separation and divorce rates first increased and then stabilized or decreased with union duration, and separation risks were higher during the first years of cohabitation than during the first years of marriage. Both separation and divorce rates increased across cohorts and decreased with the woman’s age, and the age gradient strengthened for recent cohorts. Pregnancy increased the risk of entry into cohabitation and, even more so, the risk of marriage. Although the relative effect of pregnancy on entering cohabitation increased, its effect on marrying declined across cohorts. Pregnancy within cohabitation increased the risk of marriage, again diminishing across cohorts, and decreased the risk of separation. Children generally depressed the risk of marriage within cohabitation except when they were very young. Children in a partnership were associated with lower separation and divorce risks, also particularly when they were very young. These consistencies with previous research provided preliminary evidence for the validity of the simulated life courses.

Table 1 shows the resulting macro-level distribution of union status at first birth for the simulated populations of Italian, British, and Scandinavian women born in 1940–1979. These values are very close to those in the original samples for the older cohorts, whose family life trajectories are observed throughout the reproductive ages. Differences for the younger cohorts are consistent with the fact that we project events beyond the ages at which they were observed. As observational studies have shown, the percentage of births to cohabiting women is higher among younger cohorts in all three populations, but the absolute levels depend on the context. Among the simulated Italian and British cohorts of the 1940s, only 1 % to 2 % had first births in cohabitation, compared with 13 % in the Scandinavian countries. The youngest simulated birth cohort in the Italian population had about the same proportion of births in cohabitation as did Scandinavian women born in the 1940s, whereas more than one-third of their simulated counterparts in Great Britain and more than 60 % of the youngest simulated Scandinavian cohort had a first birth in cohabitation.

**Table 1:** Percentage of first births by union status at birth in simulated populations

	Mother’s Birth Cohort				
	1940–1949	1950–1959	1960–1969	1970–1979	All Cohorts
Italy					
Cohabitation	1.2	2.5	5.1	12.1	4.8
Marriage	98.8	97.5	95.0	87.9	95.2
Great Britain					
Cohabitation	2.4	6.7	20.8	34.9	15.1
Marriage	97.6	93.3	79.2	65.1	84.9
Scandinavia					
Cohabitation	12.5	29.0	55.5	62.7	40.2
Marriage	87.5	71.0	44.5	37.3	59.8

Table 2 shows for each simulated population the percentage of parents separating by union status at first birth and by mother’s birth cohort. Again, estimates are very close to the observed separations among the older cohorts, and differences for the younger cohorts are consistent with the fact that they were not observed at older ages. This holds particularly for divorce of married parents in all countries. When cohabiting first births were very rare, as in Italy, the estimated separations were slightly higher than observed rates. For the 1940s British cohort, estimates were slightly lower than observed (Winkler-Dworak et al. 2019). In the simulated populations, separation after first birth dramatically increased across the simulated birth cohorts in Italy but remained at less than one-half the rate in Great Britain and the Scandinavian countries. Except for the youngest cohort, separation rates were quite similar for Great Britain and the Scandinavian countries; the 1970–1979 birth cohort in Great Britain had a higher separation rate than its simulated Scandinavian counterpart. In each country, as expected, separation rates were much higher for those having a first birth in cohabitation than in marriage.

**Table 2:** Percentage of first birth unions ended by union status at birth in simulated populations

	Mother’s Birth Cohort				
	1940–1949	1950–1959	1960–1969	1970–1979	All Cohorts
Italy					
Cohabitation	27.1	34.1	29.0	27.6	28.8
Marriage	3.7	7.9	9.6	11.6	7.9
All births	3.9	8.5	10.6	13.5	8.9
Great Britain					
Cohabitation	35.0	38.6	49.1	57.5	51.5
Marriage	20.1	25.0	31.7	32.5	26.3
All births	20.5	25.9	35.4	41.2	30.1
Scandinavia					
Cohabitation	30.9	37.8	42.1	40.6	39.9
Marriage	19.7	26.3	30.5	22.2	24.1
All births	21.1	29.6	37.0	33.7	30.4

Increases in parental separation did not, however, occur only among those who were cohabiting at first birth. In the Italian simulated population, separation after a first birth in cohabitation remained quite stable across cohorts, with most of the increase occurring among those married at first birth. In the British simulated population, increases in separation were found among both groups of parents, as was the case in the Scandinavian population. Divorce stabilized in Great Britain for the younger cohorts. In Scandinavia, stabilization was observed among cohabiting parents, and divorce among the 1970s birth cohort declined and was about the same as in the 1940s cohort. In general, as the difference in separation rates decreases, the compositional effects on the overall increase in parental separation should weaken.

**Fig. 2:**
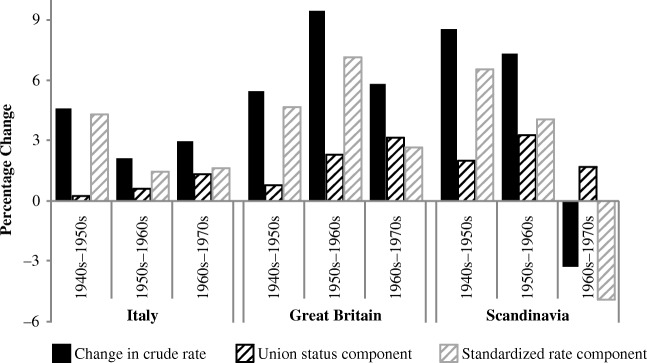
Cohort change (1940s–1970s) in parental separation: Union composition versus rate

Initial results of the decomposition analysis are presented in Fig. 2. Data for Italy are at the far left; Great Britain, in the middle; and the Scandinavian countries, at the far right. For each country, the solid black bars represent the overall change in parental separation between each pair of cohorts (1940s–1950s on the left, 1950s–1960s in the middle, 1960s–1970s on the right), simply the difference between the rates shown earlier in Table 2. The dark striped bars represent the amount of change attributable to changes in union status at birth, and the light striped bars represent the remaining change (i.e., that due to an overall change in the rate of separation).

In comparison with the 1940s cohort, Italian women born in the 1950s experienced a higher rate of parental separation that was not related to shifts in union status at birth. Most of the increase was due to increases in the overall separation rate (4.4 of the total rate change of 4.6). Composition played some role in parental separation increases for the 1960s cohort (0.6 of 2.1) and a larger role for the 1970s cohort (1.3 of 3.0).

In the British population, the overall effect of the shift toward cohabiting births accounted for only one-sixth of the increase in parental separation for the 1950s cohort (0.8 of 5.4). The dramatic increase in cohabiting first births for the 1960s cohort contributed to about one-quarter of the increase in parental separation (2.3 of 9.4). For the 1970s cohort, the increase in cohabiting first births accounted for more than one-half of the further increase in parental separation (3.2 of 5.8).

In the Scandinavian population, compositional effects played a larger role in the growth of parental separation for the earlier cohorts in comparison to the Italian and British populations, consistent with the higher levels of births in cohabitation among the older Scandinavian cohorts. About one-quarter of the increase for the 1950s cohort (2.0 of 8.6) and about one-half of the increase for the 1960s cohort (3.3 of 7.3) could be attributed to the increase in cohabiting first births. Increases in cohabiting births continued to contribute positively to parental separation for the 1970s cohort, but a sharp drop in the separation rate more than compensated such that the overall separation rate declined.

The decomposition analysis also estimates contributions of change in each union status and change in their respective rates of separation to the overall change in parental separation. Table 3 provides these estimates. First, because the union statuses are mutually exclusive, the contributions of each must be in opposing directions, cohabitation contributing to increases in separation and marriage contributing to decreases (given that parents cohabiting at first birth have higher separation rates than those married at birth). Their sum is the change in total that is attributable to changes in union status at birth (shown in Fig. 2, dark striped bars). In contrast, changes in rates of separation when first births occur in cohabitation or marriage may contribute to increases or decreases, or they may contribute in different directions to changes in the overall parental separation rate. In Italy, increases in the standardized rate of divorce contributed the most to increases in parental separation for all cohort pairs, whereas increases in the standardized rate of separation among cohabiting couples contributed virtually nothing. In Great Britain, divorce rate increases were also the biggest factor in overall increases in parental separation through the 1960s, but increases in separation rates for cohabiting parents became important in the 1970s. In the Scandinavian countries, increases in divorce rates were most important for the 1950s increase in parental separation, whereas increases in the standardized rates of divorce and cohabiting parents’ separation were both important for the 1960s increase. For the 1970s, declines in the standardized rates for both groups of parents dampened parental separation overall.

**Table 3:** Decomposition of change in probability of parental separation, union at first birth

	Mother’s Birth Cohort		
	1940s–1950s	1950s–1960s	1960s–1970s
Italy			
Change in crude rate	4.6	2.1	3.0
Change due to shift in union context	0.3	0.6	1.3
Cohabiting	0.3	0.8	2.0
Married	0.0	–0.2	–0.7
Change due to change in standardized rate	4.4	1.5	1.6
Cohabiting	0.2	–0.2	–0.1
Married	4.1	1.7	1.8
Great Britain			
Change in crude rate	5.4	9.4	5.8
Change due to shift in union context	0.8	2.3	3.2
Cohabiting	1.6	6.2	7.4
Married	–0.8	–3.9	–4.2
Change due to change in standardized rate	4.7	7.1	2.7
Cohabiting	0.2	1.5	2.4
Married	4.5	5.6	0.2
Scandinavia			
Change in crude rate	8.6	7.3	–3.3
Change due to shift in union context	2.0	3.3	1.7
Cohabiting	5.3	10.4	2.7
Married	–3.3	–7.1	–1.0
Change due to change in standardized rate	6.6	4.0	–4.9
Cohabiting	1.8	2.0	–0.6
Married	4.7	2.0	–4.3

**Fig. 3:**
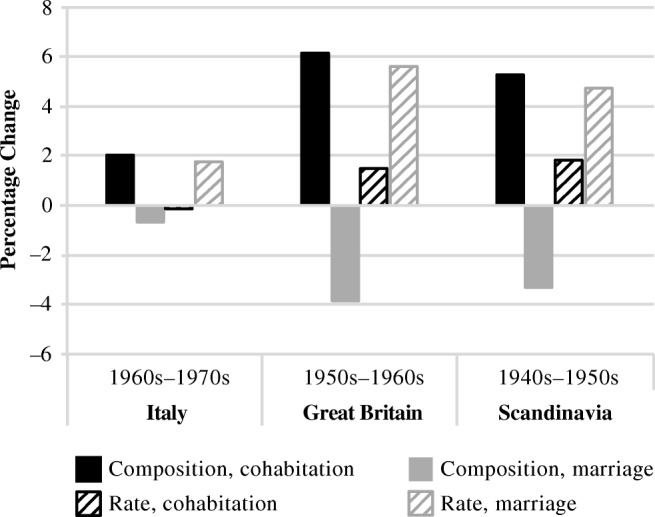
Change in parental separation specific to the cohorts in which cohabiting parenthood took off: Contributions of union composition and separation rates

In Fig. 3, we illustrate a key feature of these results: the changes between birth cohorts that experienced the take-off of cohabiting births in each country. This is the point when we might expect compositional effects to be large if the difference in separation rates for married and cohabiting parents is relatively stable. Compositional effects could also be large if the take-off cohorts generated a larger difference in separation rates for cohabiting and married parents. For Italy (left), we present the decomposition for increases in parental separation between the youngest two cohorts; for Great Britain, changes between the middle two cohorts; and for Scandinavia, changes between the older two cohorts. In Fig. 3, the solid black bar represents the contribution of the proportion of births in cohabitation, and the solid gray bar shows the contribution of proportion of births in marriage to the change in parental separation across cohorts. The dark striped bar and the light striped bar represent, respectively, contributions of changes in the separation rate for cohabiting versus married parents.

For the take-off cohorts, the patterns are very similar. The shift from marital to cohabiting first births contributed considerably to increases in separation—as discussed earlier, 1.3 of 3.0 for the youngest Italian cohorts, 2.3 of 9.4 for the middle British cohorts, and 2.0 of 8.6 for the oldest Scandinavian cohorts. The larger part of the increase in parental separation for the cohabiting pioneers in each country, however, was due to increases in parental separation rates, particularly in parental divorce.

Overall, our results show that both composition and changes in rates of separation by union type at birth contributed to increases in overall parental separation from the 1940s to the 1970s birth cohorts. Composition mattered most when cohabiting parenthood took off. Increases in divorce rates mattered more when cohabitating parenthood was less common, and increases in separation rates among cohabiting parents contributed somewhat to overall increases when cohabitating parenthood had reached relatively high levels.

## Conclusions and Discussion

We began our investigation of cohabitation and parental separation by proposing two alternative mechanisms through which the macro-level association could arise from the micro-level association. First, if cohabitation is an inherently less stable form of union, more births in cohabitation would increase the risk of parental separation overall. Second, if increases in cohabiting parenthood mean that cohabiting parents are drawn from an increasingly stable set of couple relationships, separation rates among cohabitors would decline and become more similar to divorce rates among married parents. One possible consequence is that parental separation remains relatively stable. If separation increases, it would be due to increases in both parental divorce and increases in the separation of cohabiting parents. In our simulated populations, we found that the role of cohabitation in parental separation is somewhere in between and that changes in the stability of marital unions also played a significant role.

By simulating three populations with different histories and levels of births in cohabitation, we were able to identify shifts in the contribution of cohabitation to parental separation as cohabiting parenthood dramatically increased. When cohabiting births were under 10 % (Italy for cohorts born before 1970 and Great Britain for cohorts born before 1960), the shift of births from marriage to cohabitation was less important for increasing parental separation than the increase in parental divorce rates. For cohorts experiencing dramatic increases in cohabiting parenthood (1940s–1950s in Scandinavia, 1950s–1960s in Great Britain, and 1960s–1970s in Italy), the status of parental unions at birth accounted for one-quarter to one-half of the increase in parental separation. For the youngest Italian cohort, where cohabiting births had reached only 12 %, parental divorce continued to increase and generated a large share of the increase in parental separation overall. In Great Britain, parental divorce leveled off, and separation rates among cohabiting parents increased. Thus, both the higher number of cohabiting parents and their increasing rates of separation generated very large increases in parental separation overall. In Scandinavia, cohabiting parents’ separation rates decreased but not nearly as much as divorce rates among the married parents. Even though cohabiting births continued to increase, they had smaller impacts on the overall separation rate.

Although we are mindful of the danger of “reading history sideways” (Thornton 2005), we think it is instructive to consider the maternal cohorts across countries as a single continuum with a common scenario for change in cohabiting parenthood and change in parental separation. When both phenomena emerge, as between the 1940s and 1950s Italian cohorts, parental separation is driven as much or more by divorce of the more conventional married parents as by the instability of the rare couples who do not marry before birth. When cohabiting parenthood becomes more visible—say, 10 % to 30 % of births (Italy’s 1970s cohort, Great Britain’s 1960s cohort, and Scandinavia’s 1940s and 1950s cohorts)—cohabiting parenthood becomes a larger component of the equation, but continued increases in parental divorce also contribute to increasing parental separation rates. When cohabiting births exceed 30 % (Great Britain’s 1970s cohorts, Scandinavia’s 1960s cohort), the higher separation rates of cohabiting couples begin to play a greater role than marital divorce. When most couples have their first birth in cohabitation (Scandinavia’s 1970s cohort), couples having children in marriage are increasingly selected from the most stable relationships, and their decreasing divorce rates offset the combination of high proportions of less stable cohabiting unions.

Such a scenario suggests that cohabiting parenthood is something different when it is rare than when it is common. Certainly, the more people who engage in a new behavior, the less different they are from those who do not. If the primary difference between cohabiting and married parents is the quality of their relationship or their long-term commitment to raise children together, we would expect the difference in separation rates to decline as more people have children in cohabitation. That is in fact what we observed (Andersson and Philipov 2002; Andersson et al. 2017). Some scholars have even suggested that the decision to marry before having children no longer indicates a different level of commitment to each other but is driven instead by deeply held religious convictions or “fashion” (Ohlsson-Wijk 2011). Only the former would produce increasing stability among married parents. The fact that the relative separation rates for married and cohabiting parents remained quite stable across British and Scandinavian cohorts (being higher only in Italy when cohabiting parenthood was extremely rare) suggests, however, that the decision to marry before having children remains a marker of the more stable partnerships.

The importance of parental divorce for increasing rates of parental separation was not entirely surprising but has not been highlighted in previous research on family instability. As we noted earlier, no nationally comparable statistics track divorces separately for those with and without children. Furthermore, earlier studies with survey data that did distinguish parental separation focused mainly on divorce of married parents, even where a substantial proportion of parents cohabited at birth. Research on the relative stability of cohabitation and marriage often lumps together unions with and without children. Our analysis deals with all these deficiencies to identify the role of marriage as well as cohabitation in the decreasing stability of parental unions.

The validity of our decomposition depends, of course, on the validity of the simulated populations. We checked a large number of birth and union indicators against the samples we started with and against external national data; the simulated populations appear to be valid. The greatest uncertainty is for the younger births cohorts, whose later life courses were not fully observed in the surveys but were projected relative to the experiences of older cohorts.

Further, models underlying the simulation are based only on demographic events. They incorporate (and implicitly control for) associations between cohabiting parenthood and parity, family complexity, prior cohabitation and/or marriage, and differences in the timing of births and union events across cohorts. They do not, however, incorporate variations in parental background, place of birth, education, or other experiences and characteristics that may influence life course choices. The models can be viewed as a representation of the engine of family life trajectories, with each component influenced by prior experiences and fixed characteristics.

An important distinction between our analyses and other research on parental cohabitation and separation is that we did not include marriage after birth in the decomposition. Considerable research has shown that parents who marry after birth have similar rates of divorce to those who married prior to birth (Le Bourdais and Lapierre-Adamcyk 2004; Manning et al. 2004; Musick and Michelmore 2015, 2018; Rackin and Gibson-Davis 2012; Wu and Musick 2008). The models on which these estimates are based, however, assume that a subsequent marriage risk influences the separation risk from the time of first birth. We did not make such assumptions in the regression models that underlie the simulated life courses or in the decomposition analysis. A further refinement of the decomposition could distinguish such couples as well as identify the importance of marriage timing before or after first birth for dampening the contributions of parental cohabitation at first birth to separation (cf. Holland 2013, 2017).

Our interpretation of the contribution of cohabiting parenthood to increases or leveling off of parental separation across cohorts is not that cohabitation causes separation. Rather, we interpret the decomposition as showing the extent to which the threshold of relationship commitment required for marriage—but not for parenthood—has increased. At the same time, even the decreasing proportion of those who passed the commitment threshold for marriage experienced increasing probabilities of separation (i.e., divorce). Of course, larger social and economic changes underlie the microlevel shifts in family life courses. Our analysis provides a better understanding of the mechanisms that translate those micro-level family processes into macro-level family change.

## Footnotes

1 In the text, we often use “rate,” although the strict definition in our calculations is the proportion divorced/separated among all married/cohabiting parents within a birth cohort.

2 The United States remains an outlier on parental separation at around 50 % (Andersson and Philipov 2002; Andersson et al. 2017).

3 In the version of the Harmonized Histories that we used, the ordering of union was erroneous when any of the union formation dates was missing. We reprogrammed the data to ensure that unions were in the correct order. Further, the imputation method for missing event months had been randomized across a full calendar year, producing artificial overlaps between union dates that would normally be in a different order (end of *n*th union before start of *n* + 1st union). We reversed the random imputation to fit the most likely ordering. We excluded cases where the respondent had reported all months, but two or more unions overlapped. These changes were required to appropriately assign prior births to unions in the regression models for union formation and dissolution.

4 In the hazard regressions, we checked the validity of the proportional hazard assumption and introduced interactions where necessary.
